# Immunohistochemical Characterization of Tenascin-C Expression in Treatment-Naïve Breast Cancer: An Exploratory Pilot Study

**DOI:** 10.3390/cimb48080773

**Published:** 2026-07-29

**Authors:** Kanae Taruno, Rika Narui, Sakiko Miura, Yosuke Sasaki, Miharu Kano, Toshiko Yamochi, Moriaki Kusakabe

**Affiliations:** 1 Department of Surgery, Division of Breast Surgery, Showa University School of Medicine, Shinagawa, Tokyo 142-8555, Japan; 2 Department of Pathology, Showa University School of Medicine, Shinagawa, Tokyo 142-8555, Japan; 3 Department of Diagnostic Pathology, NTT Medical Center Tokyo, Shinagawa, Tokyo 141-8625, Japan; 4 Research Center for Food Safety, Graduate School of Agricultural and Life Sciences, The University of Tokyo, Bunkyouku, Tokyo 113-0032, Japan

**Keywords:** tenascin-C, immunohistochemistry, breast cancer, tumor stroma, extracellular matrix

## Abstract

Objectives: Tenascin-C (TNC) is an extracellular matrix protein associated with tissue remodeling, tumor progression, and poor prognosis in breast cancer. Although TNC has attracted interest as a potential stromal biomarker, its baseline histopathological distribution in untreated breast cancer has not been fully characterized. This exploratory pilot study aimed to characterize the immunohistochemical distribution of TNC in representative treatment-naïve breast cancer tissues. Methods: Immunohistochemical staining for TNC was performed in representative breast cancer tissue specimens obtained from 16 treatment-naïve patients encompassing major histological and molecular subtypes. Tumor and non-tumor areas were evaluated descriptively by two board-certified pathologists using consensus assessment. Results: Heterogeneous TNC expression was observed in the stroma surrounding invasive tumors in all cases. In most specimens, TNC staining was predominantly localized to the peritumoral stroma, whereas occasional staining was observed around non-invasive lesions. Non-tumor breast stroma was largely negative, although positive staining was observed in biopsy scars and sclerotic areas. No consistent subtype-specific pattern of TNC distribution or staining intensity was identified. Conclusions: TNC showed heterogeneous stromal expression in treatment-naïve breast cancer and was also detected in biopsy scars and areas of stromal remodeling. These findings provide baseline histopathological information regarding TNC localization in untreated breast cancer. Further studies are required to determine the potential clinical utility of TNC as a stromal biomarker, including its application in the post-neoadjuvant setting.

## 1. Introduction

Breast cancer is the most common malignancy among women and the second leading cause of cancer-related deaths in the United States, according to statistics published by the American Cancer Society in 2023 [1]. The tumor microenvironment generally comprises stromal and tumor cells that produce a specialized extracellular matrix, growth factors, cytokines, and matrix remodeling enzymes. Together, these elements provide critical signaling cues that regulate tumor cell survival, proliferation, invasion, and metastasis. Extracellular matrix molecules, such as collagens, laminins, oncofetal fibronectin, and others, are highly expressed in cancerous tissues and are now recognized for their roles in promoting tumor progression [2,3,4].

Tenascin-C (TNC) is one such extracellular matrix glycoprotein that is minimally present in normal tissues but is often upregulated during tissue remodeling, such as in wound healing, inflammation, and cancer [5]. It plays key roles in regulating cell migration, proliferation, invasion, and angiogenesis. Elevated TNC expression is associated with poorer survival in glioma patients and reduced metastasis-free survival in patients with breast cancer, suggesting that TNC contributes to tumor progression [3,6]. Notably, TNC influences tumor angiogenesis by triggering the angiogenic switch, resulting in an increase in abnormal, leaky blood vessels [7]. In addition to enhancing cell migration and angiogenesis, TNC inhibits focal adhesion formation and functions as a survival factor [8]. Its expression is linked with the development and advancement of several neoplasms, including colon and breast cancer, fibrosarcoma, lung cancer, melanoma, squamous cell carcinoma, bladder cancer, and prostatic adenocarcinoma [8]. Importantly, TNC is highly expressed in high-grade gliomas, where it correlates with increased glioma cell invasiveness [9,10].

Genetic analyses have revealed that alternative splicing of nine exons in the fibronectin type III region of TNC produces multiple isoforms [11], which vary between normal and cancerous breast tissue. The fully truncated TNC isoform is more prevalent in normal and benign tissues, whereas higher molecular weight isoforms are predominantly found in cancerous tissues [11].

TNC is highly expressed in the tumor stroma, particularly in areas known as tumor matrix tracks. These regions contain various matrix molecules and are rich in fibroblasts and leukocytes [12,13]. Furthermore, TNC is found in collagen-rich regions with tumor-associated collagen signatures, which are linked to poor survival outcomes in breast cancer patients [14].

In breast cancer, TNC is one of the most prominently overexpressed extracellular matrix proteins and has been associated with poor clinical outcomes. Because anti-TNC antibodies selectively accumulate in tumor stroma, TNC has also attracted attention as a potential biomarker for tumor localization and imaging. Previous studies have suggested that stromal remodeling associated with TNC may reflect tumor progression and treatment response.

Although TNC has been proposed as a potential marker for identifying residual tumor tissue after neoadjuvant chemotherapy (NAC), the baseline histopathological distribution of TNC in untreated breast cancer has not been sufficiently characterized. A clear understanding of physiological and tumor-associated TNC localization before treatment is essential for interpreting its potential role in post-treatment specimens.

The aim of this exploratory pilot study was to characterize the immunohistochemical distribution of TNC in representative treatment-naïve breast cancer specimens across different histological and molecular subtypes. These findings provide baseline histopathological information that may support future investigations of TNC in breast cancer, including its potential application in the post-neoadjuvant setting.

## 2. Materials and Methods

### 2.1. Study Design and Participants

This single-center exploratory pilot study was conducted at Showa University Hospital, Tokyo, Japan, using breast cancer tissue specimens resected between April 2022 and October 2022. Patients who had not received preoperative systemic therapy and who had provided written informed consent for tissue storage and research use were eligible for inclusion. Representative cases were selected from the available patients during the study period to include the major histological and molecular subtypes of breast cancer. This study was designed as an exploratory histopathological investigation to characterize the baseline distribution of TNC expression across different breast cancer subtypes rather than to perform statistical comparisons between subgroups.

### 2.2. Ethics Approval

The study was approved by the Ethics Committee of Showa University, Shinagawa, Japan (approval number: M3377, 18 February 2021). This retrospective study was conducted using an opt-out approach approved by the Ethics Committee. All patients had previously provided written informed consent for the storage and research use of their tissue samples. The study was conducted in accordance with the principles of the Declaration of Helsinki. This manuscript was prepared in accordance with the REMARK guidelines.

### 2.3. Collection and Preparation of Breast Tissue Specimens

Breast tissue specimens were collected during breast cancer surgery and then prepared for immunostaining. The tissues were fixed in 10% neutral buffered formalin (Asiakizai Inc., Tokyo, Japan) immediately after collection, and the maximum fixation time was 72 h. Thereafter, the samples were embedded in paraffin, and 4 µm-thick sections were cut using a microtome (REM-700, Yamato Kohki Industrial Inc., Saitama, Japan). Hematoxylin and eosin (H&E) staining was performed to evaluate histological features before immunostaining.

### 2.4. Immunostaining

TNC expression in tumor and non-tumor areas was evaluated by immunohistochemistry using a rat monoclonal anti-human Tenascin-C antibody (Clone 3-6C2; Catalog No. 018-21781; FUJIFILM Wako Pure Chemical Corporation, Osaka, Japan). Briefly, the pathological sections were deparaffinized, placed in citric acid (pH 3.0), heated at 98 °C for 10 min, and reheated. The sections were then washed with phosphate-buffered saline (PBS; pH 7.4). The sections were then incubated in the blocking solution (Protein Block, Agilent Technologies, Santa Clara, CA, USA) at room temperature for 20 min. The blocking solution was then discarded, and sections were incubated with the primary antibody (1:200 dilution) at 37 °C for 30 min or at room temperature for 1 h. The sections were then washed with PBS, followed by incubation with the secondary antibody (Simple Stain MAX-PO (Rat); Nichirei Biosciences Inc., Tokyo, Japan). The DAB substrate kit (Nichirei Biosciences Inc., Tokyo, Japan) was used for 5 min at room temperature to develop the sections. Color development was confirmed, and the reaction was terminated. HE-stained and immunohistochemically stained slides were observed using an Olympus BX43 microscope (Olympus, Tokyo, Japan) equipped with a DP23 digital camera. Representative areas were selected manually by a pathologist, and the images were captured using cellSens Standard imaging software ver1.11 (Olympus, Tokyo, Japan). Representative tumor and non-tumor regions were jointly evaluated by two board-certified pathologists using light microscopy until consensus was reached. Stromal TNC expression was assessed semi-quantitatively according to the distribution of immunoreactivity. Weak staining was defined as focal stromal staining, whereas strong staining was defined as diffuse stromal staining. Because this study was designed as an exploratory pilot investigation, H-score, digital image analysis, and quantitative image measurements were not performed.

## 3. Results

Baseline data for the participants in this study are presented in Table 1, and TNC staining details for the 16 patients are shown in Table 2. Because this exploratory study was not designed to evaluate clinical outcomes, no further analysis of recurrence was performed.

**Figure 1:**
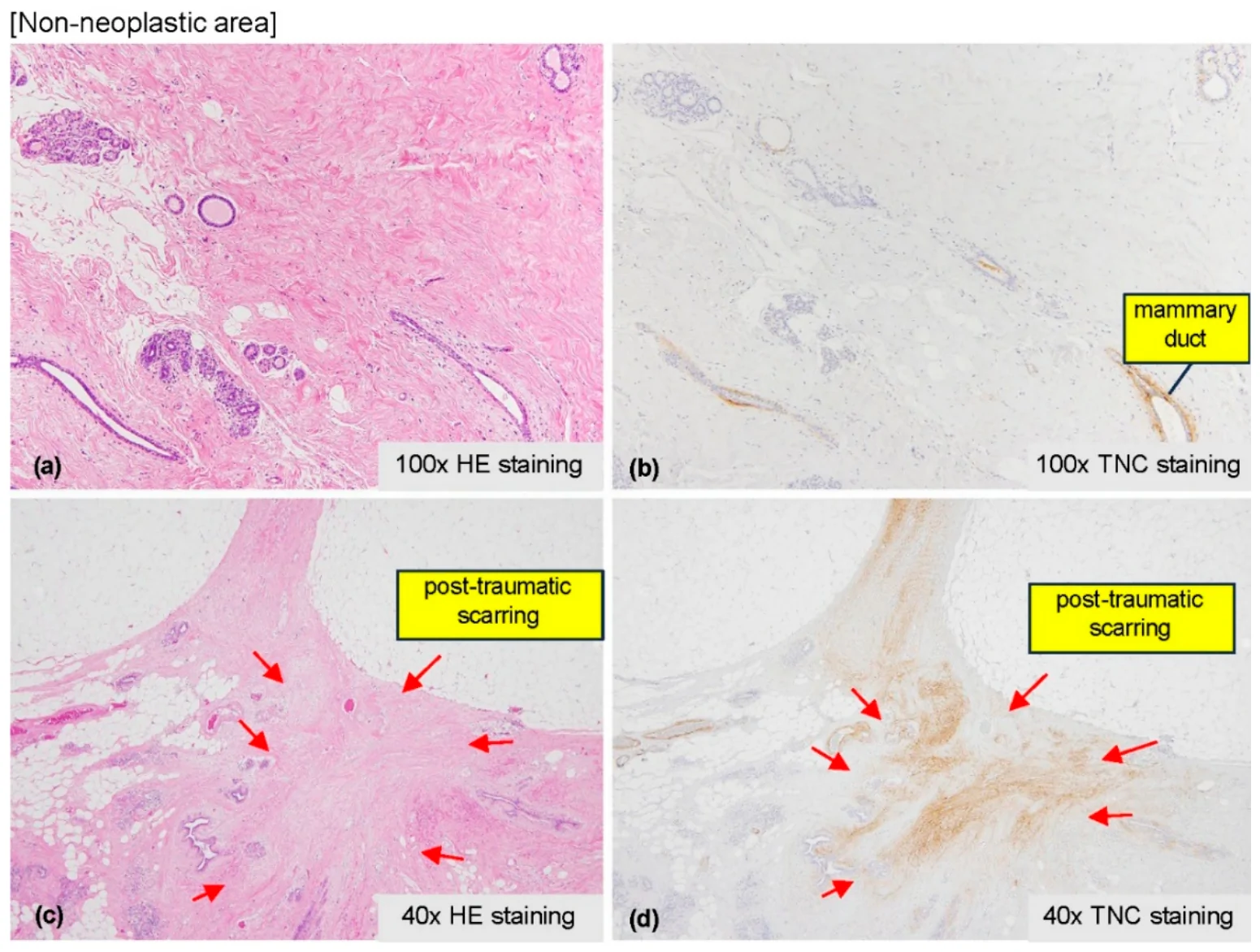
Representative non-neoplastic breast tissue. (**a**,**c**) H&E staining and (**b**,**d**) corresponding Tenascin-C (TNC) immunohistochemical staining. Minimal stromal TNC expression is observed in normal mammary tissue, whereas positive staining is observed around mammary ducts and within biopsy-associated scar tissue (arrows). Original magnifications: (**a**,**b**) ×100; (**c**,**d**) ×40.

Immunohistochemistry for TNC in the non-neoplastic areas revealed that TNC staining was largely absent in the non-tumor stroma. However, staining was observed in the mammary ducts and in biopsy-associated scar tissue (Figure 1). TNC expression was observed in the tumor-surrounding stroma in breast cancer specimens from patients who did not receive preoperative systemic therapy.

**Figure 2:**
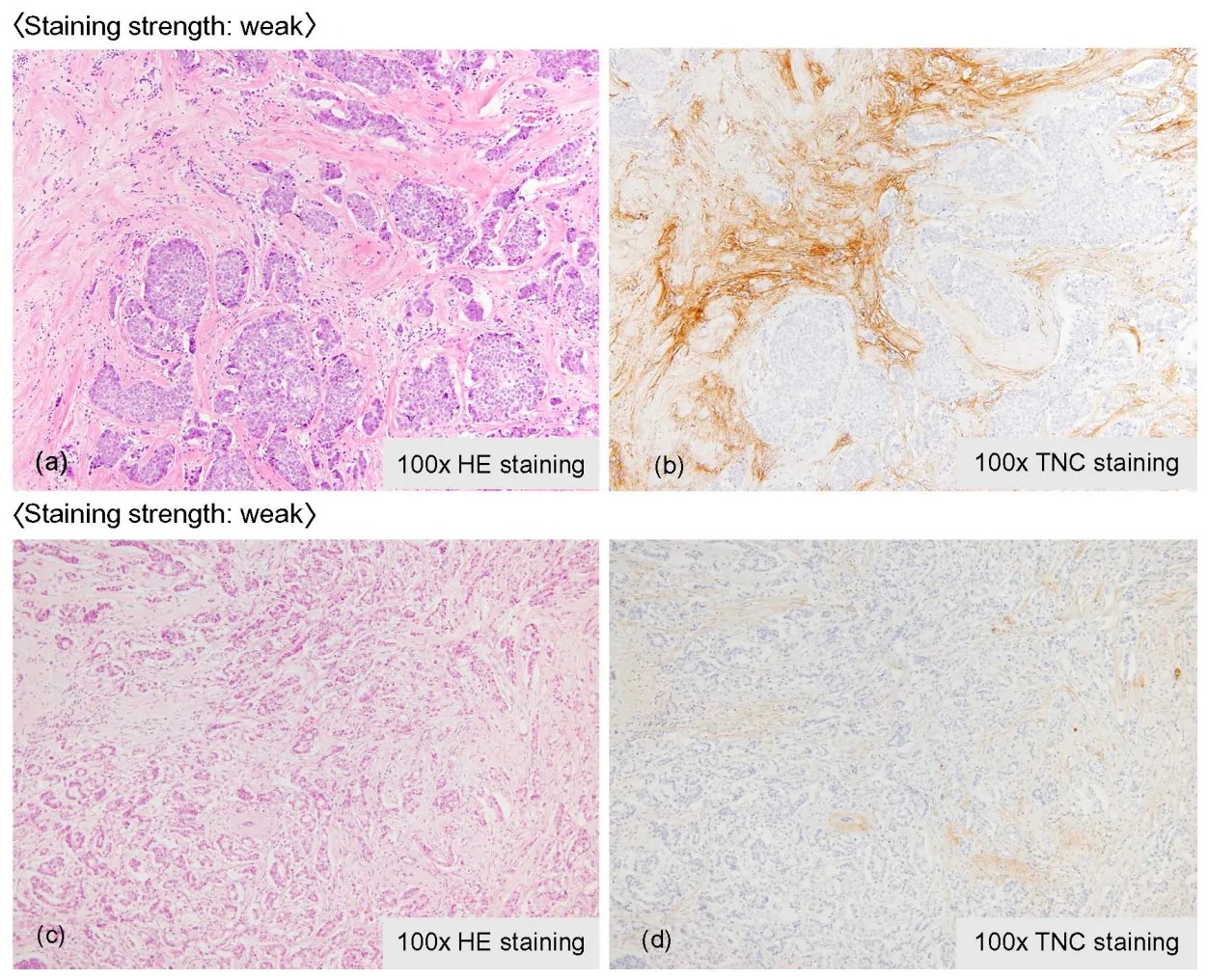
Representative invasive breast carcinoma showing weak stromal Tenascin-C (TNC) expression. (**a**,**c**) H&E staining and (**b**,**d**) corresponding TNC immunohistochemical staining from two representative cases. Focal stromal TNC expression surrounding invasive carcinoma is demonstrated in both cases. Original magnification: ×100.

Staining was observed in the peritumoral stroma of all invasive carcinomas; however, there were variations in intensity and distribution (Figure 2). No consistent subtype-specific differences in TNC distribution or staining intensity were identified across the breast cancer subtypes included in this exploratory cohort.

**Figure 3:**
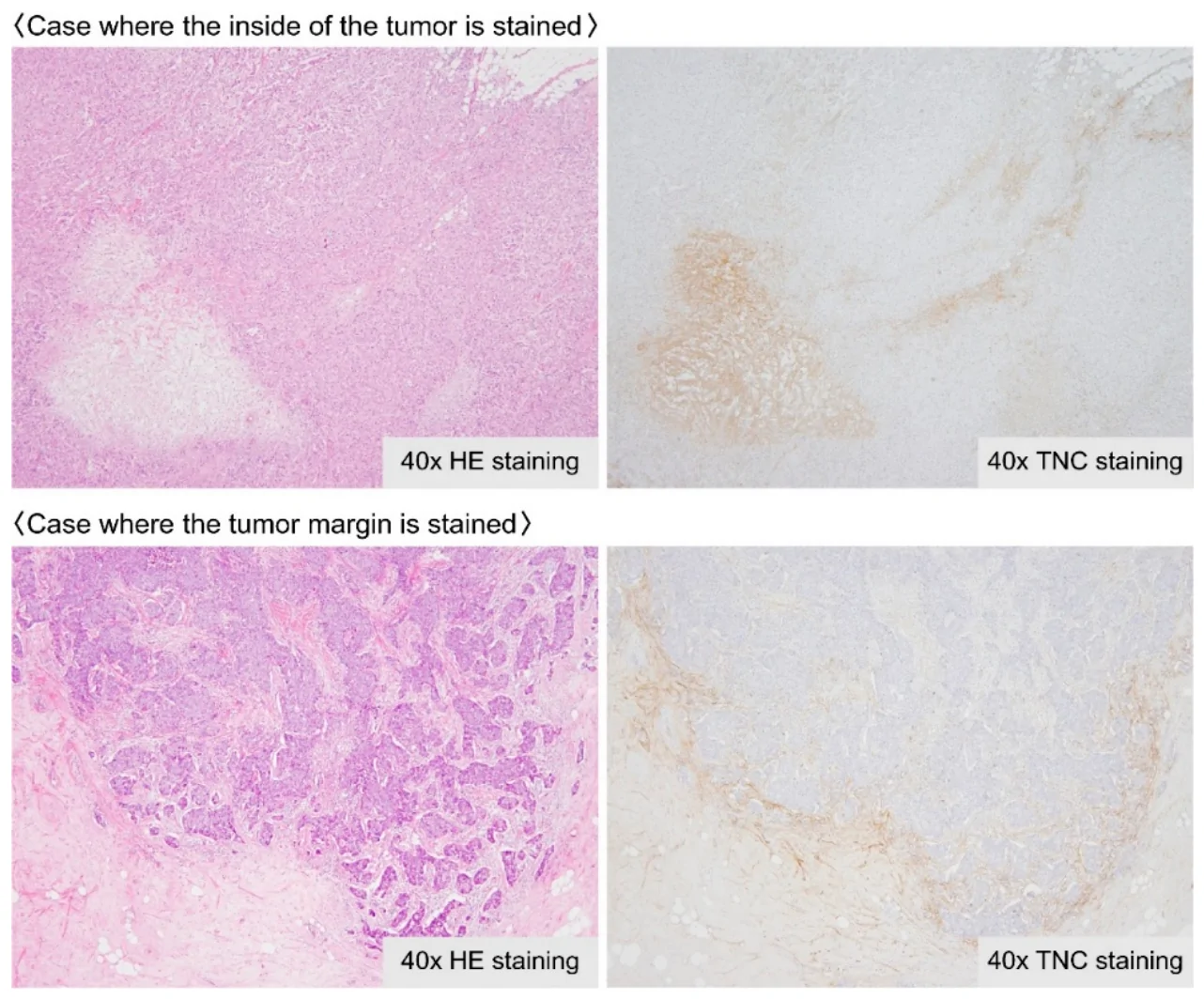
Representative patterns of stromal Tenascin-C (TNC) localization in invasive breast carcinoma. The (**upper**) panels demonstrate TNC expression predominantly within the intratumoral stroma, whereas the (**lower**) panels show stromal TNC expression predominantly at the tumor margin. Corresponding H&E staining is shown on the (**left**) and TNC immunohistochemical staining on the (**right**). Original magnification: ×40.

The localization of stromal TNC expression differed among cases. In some specimens, staining was predominantly observed within the intra-tumoral stroma, whereas in others it was more prominent at the tumor margin (Figure 3).

**Figure 4:**
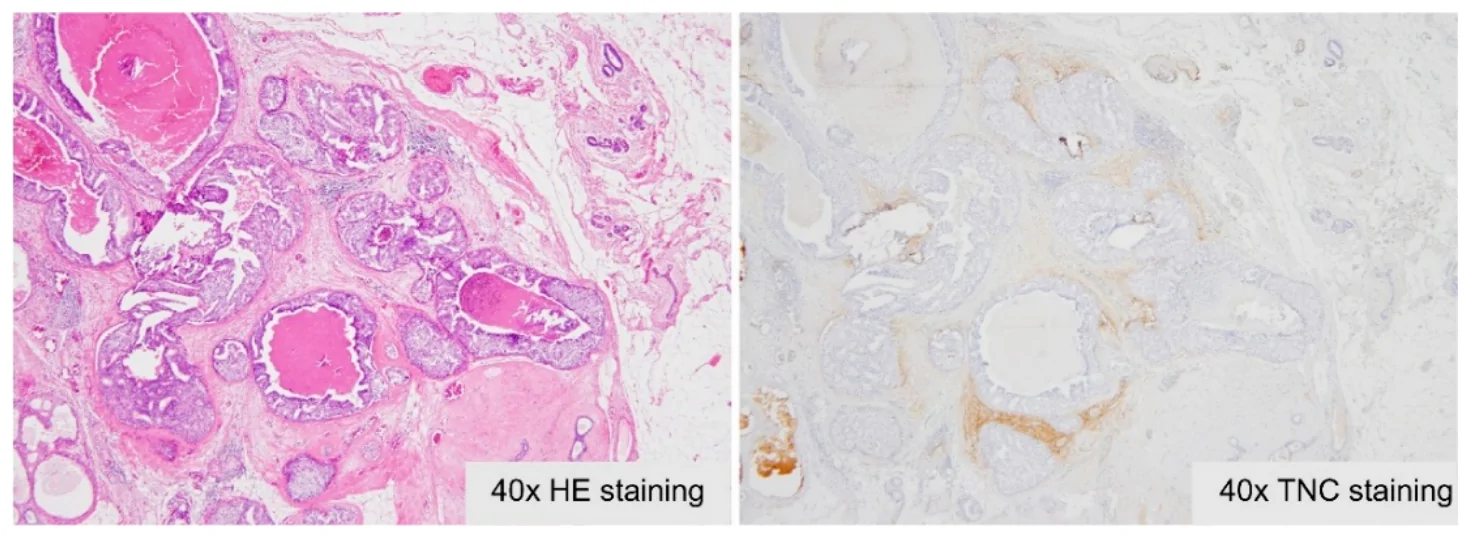
Representative ductal carcinoma in situ (DCIS) showing localized periductal stromal Tenascin-C (TNC) expression. Corresponding H&E staining is shown on the (**left**) and TNC immunohistochemical staining on the (**right**). Original magnification: ×40.

In non-invasive carcinomas, TNC staining tended to be localized to the periductal stroma surrounding the intraductal lesion, whereas invasive carcinomas showed more extensive stromal distribution (Figure 4).

Although TNC expression was observed in all patients, considerable inter-case variability was noted in both the distribution and intensity of stromal staining. No consistent subtype-specific pattern was identified in this exploratory cohort. Because this was an exploratory descriptive study, the observed staining patterns were interpreted qualitatively, and no statistical comparison between breast cancer subtypes was performed.

## 4. Discussion

Our exploratory pilot study demonstrated that TNC was consistently expressed in the stroma surrounding invasive breast cancer, although substantial inter-case variability was observed in both the intensity and distribution of stromal staining. In contrast, TNC expression was largely absent in non-neoplastic breast stroma, except in biopsy scars and areas of stromal remodeling. Non-invasive carcinomas exhibited more localized TNC staining within the periductal stroma than invasive carcinomas, while no consistent subtype-specific pattern of TNC expression was identified. Collectively, these findings provide baseline histopathological evidence regarding the localization of TNC in treatment-naïve breast cancer tissues and establish a foundation for future investigations into its potential role as a stromal biomarker in breast cancer.

There is an urgent clinical need to identify biomarkers that can predict which patients will benefit most from cytotoxic chemotherapy. Emerging evidence underscores the crucial role of intra-tumoral stroma in promoting several cancer hallmarks. In node-negative premenopausal breast cancer patients treated with a single perioperative dose of doxorubicin-based chemotherapy, tumors with high stroma content were associated with a higher risk of relapse, independent of other clinicopathological variables [15].

In breast cancer, elevated TNC expression is associated with poor prognosis. Moreover, early upregulation of TNC following neoadjuvant chemotherapy has been linked to pathological lymph node involvement at the time of surgery [16]. Functional studies have shown that both THBS1 and TNC protect MCF-7 cells from docetaxel-induced growth inhibition by activating the integrin β1/mTOR pathway and disrupting normal cell cycle regulation [16]. TNC-driven activation of integrin β1/mTOR signaling may serve as a key survival mechanism, enabling breast cancer cells to resist chemotherapy. This pathway likely plays a pivotal role in the development of chemoresistance and presents a promising target for therapeutic intervention. These findings provide critical insights into the stromal contribution to chemotherapy resistance and underscore the potential of stromal biomarkers as predictors of treatment outcomes.

Wawrzyniak et al. [8] investigated the effects of TNC loss-of-function in breast cancer cells using ATN-RNA, a dsRNA molecule homologous to TNC mRNA. The impact of ATN-RNA treatment on MDA-MB-231 breast cancer cells grown in both monolayer and three-dimensional cultures was assessed. They evaluated changes in cell proliferation, migration, adhesion, cell cycle progression, multi-caspase activation, and involvement in epithelial-to-mesenchymal transition. Oncogenomic data were also analyzed to assess the clinical relevance of TNC expression, revealing a correlation between TNC overexpression and poor survival outcomes in patients with breast cancer. Originally developed for brain tumors, the ATN-RNA approach was shown to effectively reduce the proliferation and migration of breast cancer cells and promote a shift from a mesenchymal to an epithelial phenotype. These findings suggest that TNC may serve as a universal therapeutic target in cancers where its overexpression correlates with poor prognosis. A study that used TNC as the target and radiation as the initial treatment observed an increase in TNC expression in irradiated pancreatic tumors. This approach could potentially deliver an additional antitumor agent to tumors during tissue repair after initial anticancer therapy [17].

A high combined expression score of five stromal proteins, thrombospondin 1, TNC, fibronectin, secreted protein acidic and rich in cysteine, and smooth muscle actin-α, in untreated baseline breast tumors was linked to shorter overall survival [16]. Moreover, their upregulation following chemotherapy was associated with poor treatment response. These results suggest that this stromal protein panel may function as both a prognostic tool to stratify patients based on clinical outcomes and a predictive biomarker for chemotherapy responsiveness in breast cancer.

Our findings demonstrate that TNC expression is closely associated with stromal remodeling in breast cancer. However, substantial variability in staining intensity and localization was observed among cases, and TNC expression was also detected in biopsy scars and non-neoplastic stromal remodeling. Therefore, the present findings do not support the use of TNC as a stand-alone marker for determining tumor extent. Rather, this exploratory pilot study provides baseline histopathological evidence regarding TNC localization in treatment-naïve breast cancer and establishes a foundation for future investigations evaluating its potential role as a stromal biomarker, particularly in post-treatment breast cancer specimens.

This study has several limitations. First, the sample size was small, and representative cases were intentionally selected to include different histological and molecular subtypes rather than consecutive patients, introducing the potential for selection bias. Second, stromal TNC expression was assessed descriptively using a consensus-based semi-quantitative approach without quantitative scoring methods such as H-score or digital image analysis. Third, only treatment-naïve breast cancer specimens were examined; therefore, the present findings cannot be directly extrapolated to post-neoadjuvant tissues. Accordingly, the results of this exploratory pilot study should be considered hypothesis-generating and require validation in larger consecutive cohorts, particularly those including post-treatment breast cancer specimens.

**Figure 5:**
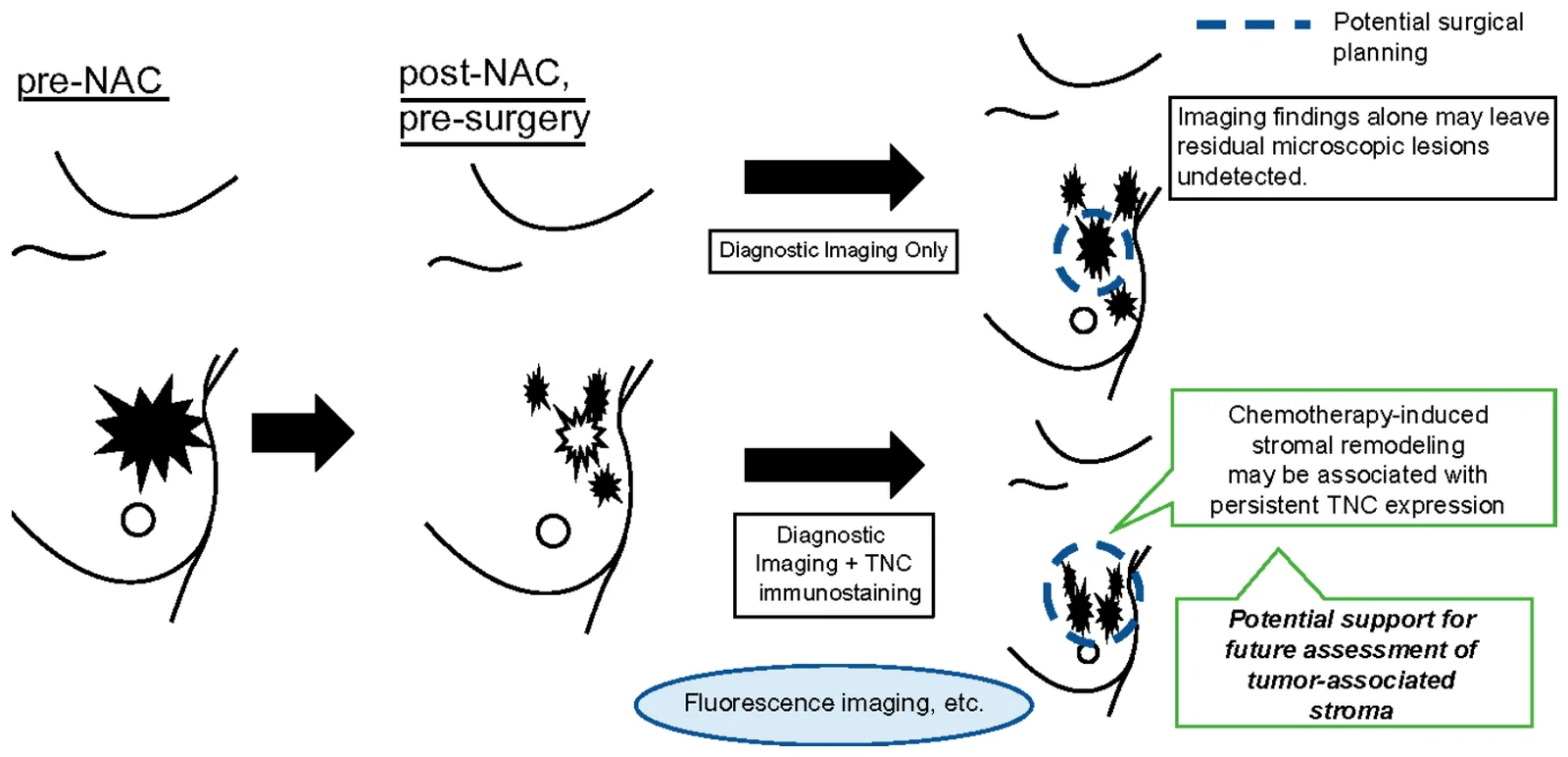
Hypothetical framework for future evaluation of Tenascin-C (TNC) as a stromal biomarker following neoadjuvant chemotherapy. This schematic illustrates a conceptual model in which persistent stromal TNC expression following chemotherapy may complement diagnostic imaging for the future assessment of tumor-associated stromal remodeling. This figure represents a hypothesis for future investigation and is not derived directly from the present experimental data.

TNC expression in stromal areas associated with tissue damage, such as biopsy scars, indicates that TNC is involved in tissue injury and repair. In high-grade tumors undergoing neoadjuvant chemotherapy, TNC might be upregulated in areas of residual tissue remodeling. The present findings generate the hypothesis that TNC may contribute to the identification of stromal remodeling following treatment. However, this concept remains speculative and requires validation using post-neoadjuvant breast cancer specimens before any clinical application can be considered (Figure 5).

## 5. Conclusions

TNC immunostaining demonstrated heterogeneous stromal localization in treatment-naïve breast cancer and provides baseline histopathological information regarding its distribution within the breast tumor microenvironment. Although TNC expression was also observed in biopsy scars and areas of stromal remodeling, these findings indicate that TNC should not currently be regarded as a stand-alone biomarker for determining tumor extent. Rather, this exploratory pilot study establishes a foundation for future investigations evaluating the potential role of TNC as a stromal biomarker, particularly in post-neoadjuvant breast cancer. Validation in larger consecutive cohorts will be required before clinical application can be considered.

## Figures and Tables

**Table 1:** Baseline data of participants.

Parameter		Number of Patients
Age, years [median (range)]		69 (38–81)
pT	0	2
	1	7
	2	5
	3	2
	4	0
pN	0	7
	1	7
	2	0
	3	2
pStage	0	2
	1	2
	2	9
	3	2
Type	Invasive ductal carcinoma	11
	Ductal carcinoma in situ	2
	Special type	3
Subtype (invasive)	Luminal A	4
	Luminal B	3
	Luminal HER2	2
	HER2	3
	Triple-negative breast cancer	2
Surgery	Mastectomy	10
	Partial mastectomy	6

**Table 2:** Details of Tenascin-C staining in 16 patients.

	Tissue Type	Subtype	Tumor Area Stromal Staining	Non-Tumor Area Stromal Staining
1	DCIS	Luminal A	Weak	None
2	DCIS	Luminal A	Weak	None
3	IDC	Luminal A	Weak	None
4	IDC	Luminal A	Weak	None
5	IDC	Luminal B	Strong	None
6	IDC	Luminal B	Weak	None
7	IDC	Luminal B	Weak	None
8	IDC	Luminal HER2	Weak	None
9	IDC	Luminal HER2	Weak	None
10	IDC	HER2	Weak	None
11	IDC	HER2	Strong	None
12	IDC	HER2	Weak, Strong	None
13	IDC	TN	Weak	None
14	ILC	Luminal A	Strong	None
15	Mucinous carcinoma	Luminal B	Weak, Weak	None
16	Apocrine carcinoma	TN	Strong	None

Cases described as “Weak, Strong” indicate heterogeneous stromal staining within the same specimen, with focal weak staining in some stromal regions and diffuse strong staining in others. Cases described as “Weak, Weak” indicate focal weak stromal staining observed in both the intra-tumoral and tumor-margin stromal regions.

## Data Availability

The datasets generated during and/or analysed during the current study are not publicly available due to Perspectives on the Protection of Personal Information but are available from the corresponding author on reasonable request.

## Abbreviations

The following abbreviations are used in this manuscript:

PBS	Phosphate-buffered saline
TNC	Tenascin-C
